# Ultrasound-Assisted Preparation of Maillard Reaction Products Derived from Hydrolyzed Soybean Meal with Meaty Flavor in an Oil-In-Water System

**DOI:** 10.3390/molecules27217236

**Published:** 2022-10-25

**Authors:** Yongkang Ye, Shengquan Dai, Hongyan Zhang, Shudong He, Wanwan Hu, Xiaodong Cao, Zhaojun Wei

**Affiliations:** 1 School of Food Science and Biological Engineering, Hefei University of Technology, Hefei 230009, China; 2 Collaborative Innovation Center for Food Production and Safety, School of Biological Science and Engineering, North Minzu University, Yinchuan 750021, China; 3 Huangshan Chaogang Food Co., Ltd., Huangshan 245000, China

**Keywords:** Maillard reaction, ultrasound, hydrolyzed soybean meal, oxidized lard

## Abstract

In the present work, we prepared Maillard reaction products (MRPs) derived from enzyme hydrolyzed soybean meal with ultrasound assistance in an oil-(oxidized lard)-in-water system (UEL-MRPs) or oil-free system (UN-MRPs), and the effect of ultrasound on the properties of the obtained MRPs was evaluated. The analysis of fatty acids in lard with different treatments showed that ultrasound can generate more unsaturated fatty acids in the aqueous phase. The UV–Vis absorbances of UEL-MRPs, UN-MRPs, and MRPs obtained in an oil-in-water system (EL-MRPs) and MRPs obtained in an oil-free system (N-MRPs) at 294 and 420 nm indicated that ultrasound could increase the amount of Maillard reaction intermediates and melanoids in the final products of the Maillard reaction. This was in line with the result obtained from color change determination—that ultrasound can darken the resultant MRPs. Volatile analysis showed ultrasound can not only increase the number of volatile substances, but also greatly increase the composition of volatile substances in UEL-MRPs and UN-MRPs, especially the composition of those contributing to the flavor of the MRPs, such as oxygen-containing heterocycles, sulfur-containing compounds, and nitrogen-containing heterocycles. Descriptive sensory evaluation revealed that UN-MRPs and UEL-MRPs had the highest scores in total acceptance, ranking in the top two, and UEL-MRPs had the strongest meaty flavor among these four kinds of MRPs. Furthermore, the measurements of antioxidant activities, including DPPH radical-scavenging activity, hydroxyl radical scavenging ability, and ferric ion reducing antioxidant power, were conducted, showing that UN-MRPs exhibited the highest antioxidant activity among all the MRPs.

## 1. Introduction

Maillard reaction products (MRPs) have a great influence on food flavor. It can not only change the flavor and color of food [1], but also impart or enhance other properties of food, such as the antioxidant and antibacterial capacity of food [2]. There have been more and more studies on the preparation of meat flavors by using Maillard reaction [3,4]. Typically, the proteins involved in the Maillard reaction system are plant-derived proteins [5] and animal-derived proteins [1,6]. However, the meat flavor produced by only using plant-derived proteins in the Maillard reaction system has obvious deficiencies (such as light meat aroma) in meat flavor. Although the meat flavor prepared by the participation of animal-derived proteins in Maillard reaction has a strong meat flavor, the pretreatment process of animal-derived proteins is often complicated [7]. Studies have shown that oxidized animal fats have a strong meaty taste. Therefore, more and more research has been focused on improving the meat flavor of MRPs by adding different animal fats into the Maillard reaction system [3,8,9,10,11]. For example, Song et al. [12] treated lard with three different lipases and investigated the effect of lard treated with different lipases on MRPs in the xylose/glucose and cysteine Maillard reaction system. They concluded that the MRPs obtained by the addition of enzymatically hydrolyzed lard had better flavor, compared with the MRPs without lard addition. Moreover, the MRPs obtained by adding lard hydrolysate treated with lipase MER had the strongest meat flavor and the lowest off-flavor. The reasons for the positive effects of fat hydrolysates on the flavor of MRPs may include two aspects. One is that the substances, such as aldehydes, ketones, alcohols, acids, and lactones, produced by fat degradation have a certain positive impact on meaty taste [8]. The other is that the carbonyl compounds produced by fat oxidation can react with amino acids and reduce sugars and their related degradation products, producing some substances with lower odor thresholds, such as aliphatic alcohols and furans, which have a positive effect on the meaty taste enhancement of MRPs [13].

Ultrasound, which has been widely used in food industry, has been conducted on promotion of Maillard reaction in recent years [14,15,16]. This is because ultrasonic waves can not only accelerate the rate of the Maillard reaction and produce more intermediates and final products, but also improve the antioxidant properties of MRPs. There are three reasons for the promotion of the Maillard reaction by ultrasonic wave. First, the cavitation effect of ultrasonic waves can generate a transient high-temperature and high-pressure environment inside the liquid, which provides extreme reaction conditions for the Maillard reaction [17]. Second, the mechanical effect of the ultrasound can accelerate the mixing of the solution and increase the frequency of intermolecular collisions, leading to the acceleration of the reaction rate [15]. Third, the activation energy required for the ultrasound-induced Maillard reaction is lower than that of conventional heat treatment [16].

Soybean meal, the by-product of soybean oil extraction, which is usually used as animal feed, possesses a high protein content of 30–50%. In order to increase its added value, there are many studies on the application of soybean meal [18,19,20], including the preparation of Maillard flavor peptides from soybean meal enzymatic hydrolysis [4,5,21]. For instance, Yu et al. [5] used peptides of different molecular weights from soybean meal hydrolysis to participate in the Maillard reaction and investigated the relationship between the antioxidant and sensory properties of the MRPs and the molecular weight of the peptides. They found that the MRPs obtained from the Maillard reaction, participating via peptides with molecular weights of 1–3 kDa, showed strongest antioxidative properties, the highest umami, and the lowest bitterness taste. In present work, we prepared MRPs using soybean meal hydrolysates and oxidized lard in an oil-in water reaction system assisted with ultrasound. The effect of ultrasonic wave treatment on MRPs was evaluated by investigating the composition and types of flavor substances, the antioxidant properties, and the sensory properties of the obtained MRPs.

## 2. Results and Discussion

This section may be divided by subheadings. It should provide a concise and precise description of the experimental results, their interpretation, and the experimental conclusions that can be drawn.

### 2.1. Fatty Acids Composition Analysis

The types of fatty acids and their content in FL, EL, and UEL that determined by GC-MS are shown in Table 1. The fatty acids detected in FL, EL, and UEL mainly included nine saturated fatty acids and eight unsaturated fatty acids. As shown in the table, the highest content of saturated fatty acids is palmitic acid (C16:0), followed by stearic acid (C18:0), and the highest contents of unsaturated fatty acids are oleic acid (C18:1) and linoleic acid (C18:2). The total content of saturated fatty acids increased slightly from 46.016 ± 0.123% in FL to 46.814 ± 0.014% in EL, and then to 46.928 ± 0.136% in UEL. The total contents in saturated fatty acids in EL and UEL are slightly higher than that in FL, which indicates that enzymatic treatment and enzymatic treatment, followed with ultrasonication, can lead to the pyrolysis and oxidation of triglycerides to form more saturated fatty acids [4]. The displayed results in Table 1 also show that the total content of unsaturated fatty acid decreased slightly from 53.984 ± 0.123% in FL to 53.186 ± 0.014% in EL, and ultimately to 53.072 ± 0.136% in UEL. The tendency is consistent with the study by Yu et al. [22]. The reasons for the decrease of unsaturated fatty acid content may come from two aspects: First, ultrasound creates a relatively high temperature environment, which accelerates the oxidation of unsaturated fatty acids, thus reducing the content of unsaturated fatty acids in UEL. Second, under the action of ultrasonic waves, the substances in the system are fully mixed, which makes more unsaturated fatty acids migrate from the oil phase to the water phase, resulting in a decrease in the proportion of unsaturated fatty acids in the oil phase. The results indicate that more unsaturated fatty acids in the aqueous phase would participate in the Maillard reaction, which might lead to the difference in the meat aroma of UEL-MRPs and other different MRPs.

### 2.2. Browning Intensity of the MRPs

**Figure 1:**
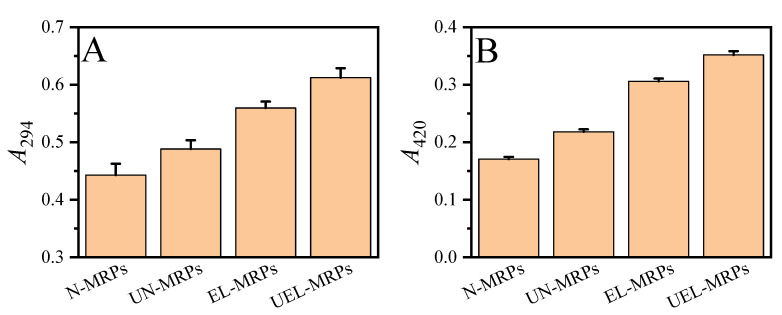
The UV–Vis absorbance at 294 nm (**A**) and 420 nm (**B**).

The degree of browning of the MRPs usually changes as the Maillard reaction proceeds. The UV–Vis absorbance at 294 nm is typically adopted to monitor the formation of Maillard reaction intermediates, while the absorbance at 420 nm is used to evaluate the brown polymer in the final products [23]. As shown in Figure 1, compared with the MRPs (N-MRPs and EL-MRPs) prepared without ultrasound assistance, the ultrasound-assisted MRPs (UN-MRPs and UEL-MRPs) exhibited higher absorbance at 294 nm (Figure 1A), and the UV absorption at 420 nm (Figure 1B) also showed the same trend as that at 294 nm. This indicates that ultrasound can accelerate the formation of Maillard reaction intermediates and increase the brown polymer in the final products. The reasons for this result may be related to two aspects. On the one hand, the mechanical effect of ultrasound can accelerate the mixing of solutions and increase the frequency of collisions between molecules [24]. This enables more Amadori compounds to degrade into Maillard reaction intermediates, which increases the absorbance of MRPs at 294 nm; at the same time, these intermediate products can be further converted into melanoid substances, thereby increasing the absorbance of MRPs at 420 nm. On the other hand, due to the cavitation effect of ultrasound, a transient high-temperature and high-pressure environment can be generated inside the liquid [17]. Compared with traditional heating procedure, ultrasound can positively promote the Maillard reaction, resulting in the production of more intermediates and melanoids, leading to higher A294/420 of the ultrasound-assisted MRPs. In addition, compared with the MRPs (N-MRPs and UN-MRPs) without lard participating in the Maillard reaction, the MRPs (EL-MRP and UEL-MRP) obtained by EL participating in the Maillard reaction showed higher absorbance at 294 and 420 nm. This may be due to the aldehydes and ketones produced by the oxidation of lard participating in the Maillard reaction, resulting in more Maillard reaction intermediates and final products [8].

### 2.3. Changes in Color

The measured values of ΔL*, Δa*, Δb* reflecting the color changes of the MRPs are shown in Table 2.

It is clear that all these parameters are negative, compared to distilled water, indicating that the color of the MRPs appears darker, greener, and bluer. In addition, compared with N-MRPs and EL-MRPs, the ΔL* values of UN-MRPs and UEL-MRPs were relatively lower. This demonstrates that the color of the MRPs obtained with ultrasound assistance were darker than those obtained without the assistance of ultrasound. This further suggests that ultrasound treatment can accelerate the Maillard reaction process, resulting in more melanoids in the MRPs [1,25]. It can also be seen from the table that the ΔL* values of EL-MRPs and UEL-MRPs were higher than those of N-MRPs and UN-MRPs. This may be due to the fact that fat degradation products participate in the Maillard reaction, producing more dicarbonyl compounds and melanoids, thus changing the color of the MRPs [3].

The ΔE* values of these MRPs are also displayed in Table 2. It is easy to find that the ΔE* value of UEL-MRPs is the largest among these MRPs, which may be related to the content of low molecular weight polymers [4,26].

### 2.4. GC–MS/SPME Analysis of the Volatile Components in the MRPs

The volatile components in the MRPs were identified by GC-MS/SPME, and the identified substances and their corresponding contents are listed in Table 3. As listed in the table, a total of 49 volatile compounds were identified in these MRPs, including 7 aldehydes, 4 ketones, 11 alcohols, 3 esters, 9 acids, 3 hydrocarbons, 4 phenols, 1 ether, 2 pyrazines, 1 pyrrole, 2 furans, 1 thiazole, and 1 thiophene. Due to the differences in the presence/absence of oxidized lard participating in the reaction and with/without the ultrasound-assisted reaction in the implementation of Maillard reaction, the types and contents of volatile components in the different MRPs were not the same. The number of the detected substances in UEL-MRPs, UN-MRPs, EL-MRPs, and N-MRPs were 35, 32, 29, and 26, respectively. Among these volatile compounds, the identified compounds, such as oxygen-containing heterocycles (furans), sulfur-containing heterocycles (thiophenes and thiazoles), and nitrogen-containing heterocycles (pyrazines and pyrroles) have a greater impact on the flavor of MRPs [4].

**Figure 2:**
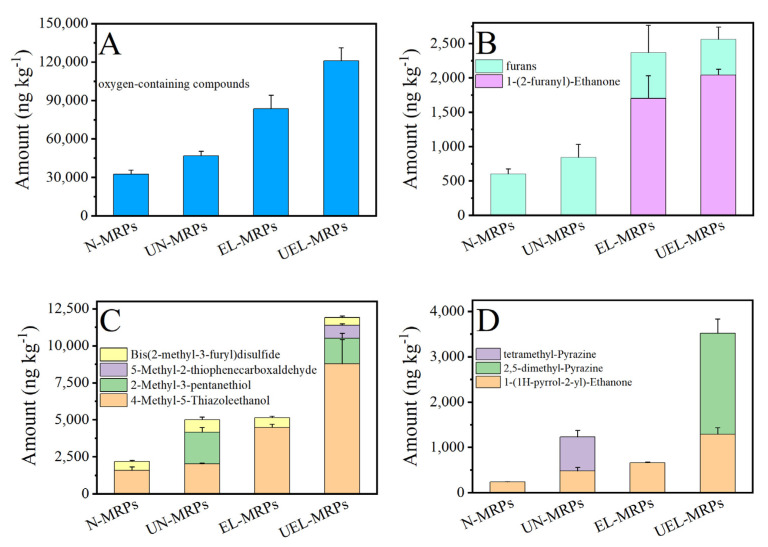
(**A**) The total amount of oxygen-containing compounds in various MRPs. (**B**) The amount of furans and 1-(2-furanyl)-ethanone in various MRPs. (**C**) The total amount of sulfur-containing compounds in various MRPs. (**D**) The total amount of nitrogen-containing compounds in various MRPs.

The histogram of the content of oxygen-containing compounds in these MRPs is shown in Figure 2A. As shown in the figure, oxygen-containing compounds are the most abundant in the types and contents of volatile compounds in all MRPs. Moreover, the oxygen-containing volatile compounds contents in EL-MRP and UEL-MRP were significantly higher than that in N-MRPs and UN-MRPs. This suggests that the participation of oxidized lard in the hydrolyzed soybean-based Maillard reaction system can significantly increase the content of oxygen-containing compounds in MRPs volatile compounds, which is similar to the results of the reported research [3]. In addition, the contents of oxygen-containing volatile compounds in UN-MRPs and UEL-MRPs were higher than those in N-MRPs and EL-MRPs, respectively. This may be related to the degree of Maillard reaction.

Furans, a class of oxygen-containing volatile compounds, have a greater impact on the flavor of MRPs [27]. Figure 2B displays the amounts of the identified furans (light cyan histogram) and a specific compound 1-(2-furanyl)-ethanone (magenta histogram) in the MRPs, respectively. It is obvious that the total amount of furans increased in the order of N-MRPs, UN-MRPs, EL-MRPs, and UEL-MRPs, and only 1-(2-furanyl)-ethanone was detected in EL-MRPs and UEL-MRPs. The 1-(2-furanyl)-ethanone has sweet, cocoa, almond, and caramel flavors [28], which can improve the flavor of MRPs. Furans can be generated from fatty acid oxidation or from glycerol and cysteine degradation products through a complex series of chemical reactions (cyclization, dehydration, and aldol condensation) [10]. Due to the participation of lard in the Maillard reaction, coupled with the assistance of ultrasound, the furan content in UN-MRPs, EL-MRPs, and UEL-MRPs was higher than that in N-MRPs, which might exert a positive effect on the flavor of these MRPs.

Sulfur-containing compounds generally have a lower odor threshold and show great impact on the meaty aroma of different foods [29]. As shown in Figure 2C, a total of four sulfur-containing substances were detected in these MRPs, namely 2-methyl-3-pentanethiol, 4-methyl-5-thiazoleethanol, bis(2-methyl-3-furyl)disulfide, and 5-methyl-2-thiophenecarboxaldehyde. It is obvious that the total amount of sulfur-containing compounds in UEL-MRPs (11,915.86 ng kg^−1^) was higher than that in EL-MRPs (5158.83 ng kg^−1^), and in UN-MRPs (5001.8 ng kg^−1^), it was higher than that in N-MRP (2183.81 ng kg^−1^). Moreover, the MRPs with oxidized lard participation in the Maillard reaction had higher content of sulfur-containing compounds than the MRPs obtained without the presence of oxidized lard. Another result that should be mentioned is that 5-methyl-2-thiophenecarboxaldehyde, which contributes to meat flavor [30], was found only in UEL-MRPs (873.56 ng kg^−1^). The identification and quantitation results of the sulfur-containing substances might be due to the participation of animal fat in the Maillard reaction [4] and the promotion of ultrasound to the Maillard reaction [15].

Nitrogen-containing heterocycles are another class of volatile substances contributing to the MRPs flavor. The nitrogen-containing heterocycles mostly exist in the forms of pyrazine, pyrrole, and pyridine. As shown in Figure 2D, altogether, three kinds of such substances were identified in these MRPs, namely tetramethyl-Pyrazine, 2,5-dimethyl-pyrazine, and 1-(1H-pyrrol-2-yl)-ethanone. The total contents of nitrogen-containing heterocycles in UN-MRPs (1234.19 ng kg^−1^) and UEL-MRPs (3523.7 ng kg^−1^) were higher than those in N-MRPs (237.32 ng kg^−1^) and EL-MRPs (665.75 ng kg^−1^), respectively. This may be due to the accelerated formation of nitrogen-containing compounds by using ultrasound assistance. Pyrazine generally has a nutty and cooked burnt aroma [31]. Figure 2D also shows that 2,5-dimethylpyrazine (2235.33 ng kg^−1^) and tetramethyl-Pyrazine (756.89 ng kg^−1^) were detected only in UEL-MRPs and in UN-MRP, respectively. This may be due to the fact that the activation energy values required for the synthesis of targeted pyrazine species in an ultrasound-assisted Maillard reaction model system were lower than those in heat treatment [32].

### 2.5. Sensory Evaluation

**Figure 3:**
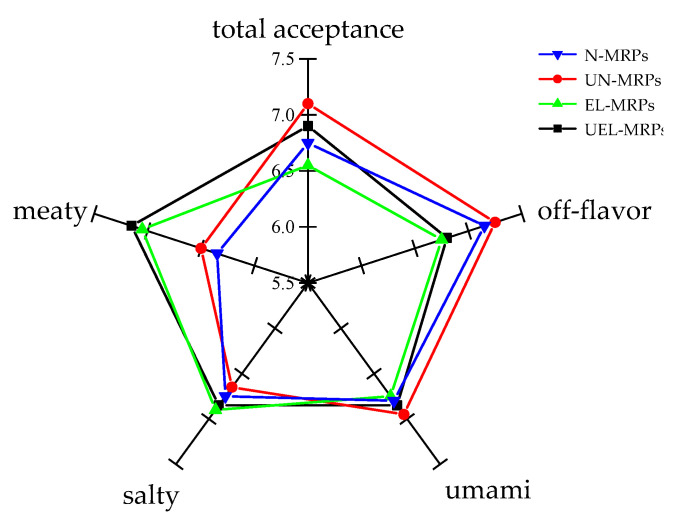
Sensory characteristics of various MRPs.

The sensory scoring criteria are listed in Table 4, and the sensory evaluation results of the MRPs are shown in Figure 3. It is clear that the participation of oxidized lard in the Maillard reaction and the application of ultrasound made little difference, regarding the salty and umami taste of these MRPs. The meaty taste of EL-MRPs and UEL-MRPs obtained from oxidized lard participation in the Maillard reaction showed better meat flavor than that of the N-MRPs and UN-MRPs obtained from the absence of oxidized lard in the Maillard reaction. In addition, the meat taste scores of the UEL-MRPs and UN-MRPs obtained by the promotion of ultrasound to the Maillard reaction were also higher than those of the EL-MRPs and N-MRPs, respectively. These may be related to their higher content of sulfur-containing compounds and nitrogen-containing heterocycles, such as thiophene and thiazole [13]. However, oxidized lard developed some undesired flavors during oxidation, which made EL-MRP and UEL-MRP have a heavier off-flavor. Compared with the MRPs of N-MRP and EL-MRP, UN-MRP and UEL-MRP had higher scores, in terms of total acceptance. The sensory evaluation results indicate that the flavor of MRPs could be improved by the application of ultrasound.

### 2.6. Sensory Evaluation

**Figure 4:**
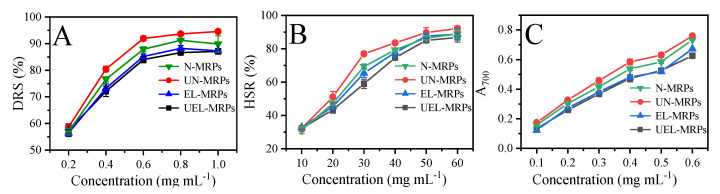
Antioxidant activity of various MRPs. (**A**) DPPH free radical scavenging activity. (**B**) Hydroxyl radical scavenging ability. (**C**) Reducing power, expressed in absorbance read at 700 nm.

The antioxidant ability of the MRPs was evaluated by measuring the DRS, HSR, and ferric ion reducing antioxidant power. Figure 4A shows the DPPH radical scavenging ability of the MRPs. Under the same condition, the scavenging rate of DPPH radicals by UN-MRPs was the strongest among the four MRPs, and the scavenging rate reached a peak value of about 94.58%, at a concentration of 1 mg mL^−1^. Furthermore, basically the scavenging rate of the DPPH radicals by these MRPs was in the order of UN-MRPs > N-MRPs > EL-MRPs > UEL-MRPs. As can be seen in Figure 4B,C, the hydroxyl radical scavenging ability (Figure 4B) and ferric ion reducing antioxidant power (Figure 4C) of the MRPs showed similar trends to the scavenging rates for DPPH radicals.

According to the antioxidant analysis, we can find that UN-MRPs showed the best antioxidant ability among these MRPs. This may be because the ultrasound-assisted heat treatment accelerates the Maillard reaction, thereby generating more substances with antioxidant capacity [1]. It has been reported that the produced intermediate pyrroles and melanoids in the final products of the Maillard reaction have the antioxidant capacity [15,33]. According to the results of the browning intensity measurement and SPME/GC-MS, more intermediate substances, melanoids, and nitrogen-containing heterocycles were found in the UEL-MRPs. However, the antioxidant capacity of UEL-MRP was measured to be the lowest among these MRPs, and that of EL-MRPs was the second lowest. Studies have shown that fragments of 30–50 kDa in the MRPs have high antioxidant activity [34,35]. The possible reason for the difference in the antioxidant activity of the MRPs may be due to the distribution of the molecular weights in these MRPs. Future work will focus on obtaining MRPs with different molecular weights and evaluating their antioxidant activity, thereby finding out the relationship between the molecular weight and antioxidant activity.

## 3. Materials and Methods

### 3.1. Materials and Chemicals

Soybean meal was purchased from Muge Feed Co., Ltd. (Hebei, China). Lard was from Yusheng Edible Oil Co., Ltd. (Shandong, China). Lipase MER (7500 Lu g^−1^) was purchased from Tianye Enzyme Preparation Co., Ltd. (Jiangsu, China). Alkaline protease (200,000 U g^−1^), neutral protease (100,000 U g^−1^), and flavor protease (200,000 U g^−1^) were obtained from Shanghai Yuanye Biotechnology Co., Ltd. (Shanghai, China). L-cysteine, D-xylose, and thiamine were purchased from Shanghai Aladdin Biochemical Technology Co., Ltd. (Shanghai, China). Other chemicals were analytical reagents and purchased from Shanghai Mackin Biochemical Co., Ltd. (Shanghai, China). Deionized water was used throughout the experiment.

### 3.2. Sample Preparation

#### 3.2.1. Preparation of Enzymatic Hydrolyzed Lard

Lipase MER was selected to prepared hydrolyzed lard [12]. Fresh lard (FL) and phosphate buffer (pH = 6.0) were mixed thoroughly at a ratio of 1:1 (*w*/*w*) and then added into a round-bottomed flask, followed with the addition of lipase MER (1 g enzyme per 100 g lard). The enzymatic hydrolysis was carried out at 45 °C for 1.5 h with magnetic stirring at a speed of 150 rmp. The enzymatic hydrolysis solution was then heated to 95 °C for 15 min to inactivate the lipase MER. The resultant solution was cooled down to room temperature, followed with centrifugation at 4000 rpm for 15 min. The supernatant solution, denoted as EL, was collected and kept at −20 °C for later use. The EL solution, treated with ultrasonication, was prepared by putting the probe of the ultrasound device (JY98-IIIDN, Ningbo Xinzhi Biotechnology Co., Ltd., Ningbo, China) 1.5 cm below the liquid level of the above collected supernatant solution (mixed solution of soybean meal enzymatic hydrolyzate, xylose, cysteine, and EF). The solution was sonicated at 300 W (5 s-on-5 s-off) for 30 min, and the obtained treated lard was named UEL. The acid values (AV) of FL, EL, and UEL were measured to be 0.48 ± 0.04, 61.84 ± 3.62, and 69.60 ± 1.73 mg g^−1^, respectively; the peroxide values (PV) were 0.080 ± 0.004, 0.085 ± 0.003, and 0.084 ± 0.005 meq. kg^−1^, respectively; and the p-anisidine values (p-AV) were 3.77 ± 0.43, 5.13 ± 0.53, and 6.04 ± 0.69, respectively.

#### 3.2.2. Preparation of Soybean Meal Hydrolysates

Soybean meal hydrolysates were prepared according to the method of Zhang et al. [21], with tiny modifications. Soybean meal (47.8% of protein content) was pulverized and sieved with a 100-mesh sieve. The soybean meal powder was then dispersed in water to prepare suspension with a protein content of 4% (*w*/*w*). It was then heated to 90 °C for 30 min, cooled to room temperature, and then sonicated at 45 °C for 20 min with an ultrasonic power of 350 W. The suspension was then enzymatically hydrolyzed by a two-step enzymatic hydrolysis method. Firstly, the pH of the suspension was adjusted to 10.0, with 6.0 mol L^−1^ of NaOH, and then it was enzymatically hydrolyzed with alkaline protease (8000 U g^−1^) at 50 °C for 3.5 h. Secondly, the pH of the above mixture was adjusted to 6.5, with 6 mol L^−1^ HCl, and it was enzymatically hydrolyzed with neutral protease (8000 U g^−1^) and flavor protease (1200 U g^−1^) at 45 °C for 4 h. The enzymatic hydrolysis solution obtained in each enzymatic hydrolysis step should be inactivated at 90 °C for 15 min, and then the soybean meal enzyme hydrolysate was centrifuged at 8000 rmp for 20 min. Finally, the supernatant was collected and stored at −20 °C for later use. The degree of hydrolysis of soybean meal enzymolysis solution was 39.04%, measured by using ortho-phthalaldehyde method.

#### 3.2.3. Preparation of Maillard Reaction Products

The Maillard reaction system without the addition of lard was prepared by adding cysteine (2.0%), xylose (3.0%), and VB_1_ (0.05%) into the soybean meal enzymatic hydrolysis, and the system with the addition of EL was prepared by adding cysteine (2.0%), xylose (3.0%), VB_1_ (0.05%), and EL (1.0%) into the soybean meal enzymatic hydrolysis. The prepared reaction solution was mixed thoroughly by stirring at 50 °C for 10 min, with a stirring speed of 150 rpm. The preparation of MRPs with the assistance of ultrasound was carried out in accordance with the following steps. First, the pH of the reaction solution was adjusted to 7.1. Next, it was sonicated by putting the probe of the ultrasound device 1.5 cm below the liquid level. The solution was sonicated at 300 W (5 s-on-5 s-off) for 30 min. The Maillard reaction was performed by maintaining the above solution at 120 °C for 2 h, with constantly stirring at a speed of 200 rpm. Finally, the obtained MRPs were rapidly cooled in an ice-water bath, and then freeze-dried to prepare a lyophilized powder, which was stored at −20 °C for later use. The obtained MRPs, with and without the addition of EL, were named as UEL-MRPs and UN-MRP, respectively.

The procedure for the preparation of MRPs without ultrasound assistance was similar to the MRPs prepared with the assistance of ultrasound, except that no sonication step was required. The obtained MRPs with and without the addition of EL were named EL-MRPs and N-MRP, respectively.

### 3.3. Analysis Methods

#### 3.3.1. Analysis of the Fatty Acid Composition in Various Lard

The fatty acid compositions in FL, EL, and UEL were analyzed using gas chromatography (GC-2010 PRO, Excellence in Science, Inc, Tokyo, Japan), equipped with a chromatographic column of SP-2560 (100 m × 0.25 mm × 0.20 μm). Before analyzing, FL, EL, and UEL were methylated using methanol containing 1 mol L^−1^ NaOH. Nitrogen was used as the carrier gas, and the gas flow was 1.0 mL min^−1^. The temperature program settings were in accordance with the method of Ye et al. [4].

#### 3.3.2. Determination of Browning Intensity and Color of MRPs

The browning intensity of these MRPs was measured on a UV-4802 UV–Vis spectrophotometer (Shanghai Unico Instrument Co., Ltd., Shanghai, China). The MRPs were diluted 150-fold and 60-fold with distilled water, respectively, and the absorbance values were measured at 294 nm (150-fold) and 420 nm (60-fold) using a UV-4802 UV–Vis spectrophotometer.

The color of these MRPs was determined by measuring the Commission International Eclairage (CIE) of lightness (*L*), parameter *a* (redness or greenness), and parameter *b* (yellowness or blueness). The measurements were performed on a NR200 portable colorimeter (Shenzhen, China) by recording ΔL*, Δa*, and Δb*, and the total color difference (Δ*E*) was calculated according to the following equation [3]:

(1) $$\Delta E=\sqrt{{\left(\Delta L^{*}\right)}^{2}+{\left(\Delta a^{*}\right)}^{2}+{\left(\Delta b^{*}\right)}^{2}}$$

#### 3.3.3. Determination of DPPH Radical-Scavenging Activity

The DPPH radical-scavenging ability of MRPs was determined according to the method of Yu et al. [5] and Zeng et al. [36], with slight modifications. Typically, 100 µL of the MRPs solution (0.2, 0.4, 0.6, 0.8, and 1.0 mg mL^−1^) and 100 µL DPPH (0.2 mmol L^−1^ in ethanol) solution were added dropwise to a 96-well microtiter plate. All these mixtures were then placed in the dark at 25 °C for 10 min. The absorbance of each sample was measured at 517 nm on a microplate reader (BioTek Instruments, Inc, Winooski, VT, USA), and the value was defined as *A_s_.* Meanwhile, the absorbance of ethanol (200 µL) and the mixture of ethanol (100 µL) and the MRPs (100 µL) were also recorded, and the values were defined as *A_c_* (absorbance of control) and *A_b_* (absorbance of blank). The DPPH radical-scavenging activity (DRS%) of the MRPs was then calculated according to Equation (2), as follows:

(2) $$\mathrm{DRS}\%=(1-\frac{A_{s}-A_{c}}{A_{b}})\;\times \;100$$

#### 3.3.4. Determination of Hydroxyl Radical Scavenging Ability

The determination of hydroxyl radical scavenging ability of the MRPs referred to the method described by Li et al. [37]. Briefly, 100 µL of the MRPs with different concentrations (10, 20, 30, 40, 50, and 60 mg mL^−1^), 100 µL of salicylic acid-ethanol solution (10 mmol L^−1^), 100 µL of FeSO_4_ (10 mmol L^−1^) solution, 700 µL of distilled water, and 1.0 mL of hydrogen peroxide solution (100 mmol L^−1^) were thoroughly mixed and incubated at 37 °C for 15 min. Then, 250 µL of the above solution was added into a 96-well microtiter plate, and the absorbance of the sample was measured at 510 nm with a microplate reader. The control and blank samples were prepared by replacing salicylic acid-ethanol and the MRPs sample with ultrapure water, respectively. The hydroxyl radical scavenging capacity (HRS%) was calculated, according to Equation (3), as:

(3) $$\mathrm{HRS}\%=(1-\frac{A_{s}-A_{c}}{A_{b}})\;\times \;100$$

where *A_s_*, *A_c_*, and *A_b_* are the absorbance of the sample, control, and blank sample, respectively.

#### 3.3.5. Determination of Ferric Ion Reducing Ability

The reducing ability of MRPs was evaluated with reference to the method of Habinshuti et al. [2]. A volume of 2.0 mL of the MRPs with various concentrations (0.1, 0.2, 0.3, 0.4, 0.5, and 0.6 mg mL^−1^), 2.5 mL of phosphate buffer saline (PBS, 0.2 mol L^−1^, pH = 6.6), and 2.5 mL of K_3_Fe(CN)_6_ solution (1.0%, *w*/*v*) were mixed and incubated at 50 °C for 20 min. Then, 2.5 mL of trichloroacetic acid solution (10%, *w*/*v*) was added into the above mixture, followed by centrifugation at 3000 rpm for 10 min at room temperature, and the supernatant was collected. Afterwards, the supernatant (2.5 mL) was added with 0.5 mL of FeCl_3_ solution (0.1%, *w*/*v*) and 2.5 mL of ultrapure water. After reacting at room temperature for 10 min, 250 µL of the mixture was added into a 96-well microtiter plate. The absorbance of the sample was measured at 700 nm on a microplate reader. The increase of the absorbance at 700 nm was used to evaluate the reducing ability of the MRPs.

#### 3.3.6. Analysis of Volatile Compounds by GC–MS/SPME

The analysis of volatile compounds in the MRPs was accomplished on a GC-MS instrument (QP2010SE, GK/J-0950, Shimadzu, Excellence in Science, Inc, Tokyo, Japan). A total of 3 µL of the internal standard 1,2-dichlorobenzene (50 µg mL^−1^ in methanol) and 3 mL of the MRPs were added into a 20 mL sealed headspace vial. Then, insert the needle of the SPME sampler into the headspace vial. The sample was equilibrated in the vial at 50 °C for 30 min to extract the volatile substances, while the SPME fiber (75 μm, carboxen/poly-dimethyl siloxane) was suspended above the liquid surface to absorb the extracted volatile substances in each MRP. Afterwards, the adsorbed volatiles were injected into the injection port of a GC-MS and desorbed at 250 °C for 5 min. The instrument was equipped with a DB wax column (30 m × 0.25 mm × 0.25 µm, Agilent Technology, Inc., Folsom, CA, USA) and a mass spectrometer. During the measurement, high-purity helium was used as the carrier gas, and the flow rate was 1.2 mL min^−1^. The column temperature program was set as follows: the initial temperature was kept at 40 °C for 3 min, increased to 200 °C at 5 °C min^−1^, and then increased to 230 °C at 10 °C min^−1^. The scanning range of mass spectrometric detector was in the range of 40–450 *m*/*z*.

#### 3.3.7. Descriptive Sensory Analysis of the MRPs

Sensory evaluation of MRPs was performed according to the method of Song et al. [12]. Ten experts with knowledge of flavor evaluation were selected from the Scientific Sensory Evaluation Laboratory of Hefei University of Technology to evaluate the descriptive senses of the MRPs. The evaluation panel consisted of 5 male and 5 female members. Prior to evaluation, unified standards (Table 4) for specific indicators of flavor, including meaty, off-flavor, umami, salty, and overall satisfaction, were set up by full discussion. The evaluation should be performed at room temperature (25 °C), and the sensory evaluation of each sample was repeated three times in parallel.

### 3.4. Statistical Analysis

The analysis of each sample was repeated triplicates, and the obtained data were analyzed by one-way analysis of variance (ANOVA) with SPSS version 26.0 (SPSS, Inc., Chicago, IL, USA) software. The analyzed data were presented as mean values ± standard deviations (SDs). Significance was considered at ± 5% (*p* < 0.05).

## 4. Conclusions

In conclusion, we analyzed the effect of ultrasound on the MRPs derived from hydrolyzed soybean meal in an oil-in-water system. The GC-MS analyses of the fatty acid compositions of lard obtained by different treatments showed that ultrasonic treatment not only accelerated the oxidation of lard, but also decreased its unsaturated fatty acid content. The addition of oxidized lard coupling with ultrasound assistance increased the UV absorbance at 294 nm and 420 nm, darkening the color of the obtained MRPs. Due to the effects (mechanical and cavitation effects) of ultrasound, the volatile compounds of the UN-MRPs and UEL-MRPs were significantly increased, compared to the controls of N-MRPs and EL-MRPs, respectively. More importantly, the volatile substances that contributed greatly to the flavor of the MRPs were increased in UN-MRPs and UEL-MRPs, compared to the controls of N-MRPs and EL-MRPs, respectively. The sensory evaluation also showed that ultrasound exerted positive effects on the taste of the obtained MRPs, as the total acceptance of the UN-MRPs and UEL-MRPs was better than that of the N-MRPs and EL-MRPs. Antioxidant tests showed that the UN-MRPs obtained by ultrasound assistance in oil-free system showed better antioxidant activity than the control N-MRPs, while the UEL-MRPs obtained in oil-in water system showed lower antioxidant activity than the control EL-MRPs. The future work will focus on the separation and purification of MRPs to obtain MRPs with different molecular weight ranges and to find out the relationship between the antioxidant activity, the molecular weight of the MRPs, and the effects of ultrasound.

## Figures and Tables

**Table 1:** GC results of fatty acid compositions in FL, EL, and UEL.

Fatty Acids	Percentage (%)		
	FL	EL	UEL
*Saturated fatty acid*			
C10:0 decanoic acid	0.020 ± 0.001 ^a^	0.021 ± 0.000 ^a^	0.017 ± 0.000 ^b^
C12:0 lauric acid	0.076 ± 0.000 ^a^	0.077 ± 0.003 ^a^	0.075 ± 0.000 ^a^
C14:0 myristic acid	0.898 ± 0.005 ^a^	0.906 ± 0.005 ^a^	0.904 ± 0.008 ^a^
C15:0 pentadecanoic acid	0.041 ± 0.005 ^a^	0.040 ± 0.002 ^a^	0.041 ± 0.003 ^a^
C16:0 palmitic acid	36.271 ± 0.090 ^b^	36.861 ± 0.16 ^a^	36.958 ± 0.142 ^a^
C17:0 margaric acid	0.129 ± 0.012 ^a^	0.130 ± 0.003 ^a^	0.136 ± 0.006 ^a^
C18:0 stearic acid	8.150 ± 0.020 ^c^	8.345 ± 0.026 ^b^	8.402 ± 0.005 ^a^
C21:0 n-heneicosanoic acid	0.293 ± 0.007 ^ab^	0.318 ± 0.026 ^a^	0.279 ± 0.003 ^b^
C22:0 behenic acid	0.137 ± 0.002 ^a^	0.118 ± 0.000 ^b^	0.116 ± 0.001 ^b^
Total	46.016 ± 0.123 ^b^	46.814 ± 0.014 ^a^	46.928 ± 0.136 ^a^
*Unsaturated fatty acid*			
C14:1 myristoleic acid	0.017 ± 0.001 ^a^	0.017 ± 0.000 ^a^	0.018 ± 0.005 ^a^
C16:1 palmitoleic acid	1.066 ± 0.026 ^a^	1.036 ± 0.001 ^a^	1.046 ± 0.018 ^a^
C17:1 heptadecenoic acid	0.060 ± 0.003 ^a^	0.056 ± 0.005 ^a^	0.058 ± 0.003 ^a^
C18:1 oleic acid	37.328 ± 0.103 ^a^	36.754 ± 0.018 ^b^	36.722 ± 0.135 ^b^
C18:2 linoleic acid	14.663 ± 0.027 ^a^	14.441 ± 0.004 ^b^	14.413 ± 0.009 ^b^
C20:1 eicosenoic acid	0.185 ± 0.005 ^b^	0.211 ± 0.003 ^a^	0.164 ± 0.016 ^c^
C18:3 α-linolenic acid	0.633 ± 0.003 ^a^	0.638 ± 0.015 ^a^	0.626 ± 0.001 ^a^
C20:3 carbonium	0.033 ± 0.003 ^a^	0.033 ± 0.000 ^a^	0.025 ± 0.001 ^b^
Total	53.984 ± 0.123 ^a^	53.186 ± 0.014 ^b^	53.072 ± 0.136 ^b^

Note: Results were expressed as mean value ± standard deviation (*n* = 3). Values bearing different letters (a to c) were significantly different (*p* < 0.05).

**Table 2:** Color changes of various MRPs.

Sample	ΔL*	Δa*	Δb*	ΔE*
N-MRPs	−2.61 ± 0.06 ^a^	−0.06 ± 0.02 ^a^	−2.07 ± 0.06 ^c^	3.33 ± 0.02 ^c^
UN-MRPs	−2.68 ± 0.03 ^a^	−0.14 ± 0.02 ^c^	−2.21 ± 0.01 ^d^	3.47 ± 0.02 ^b^
EL-MRPs	−3.42 ± 0.08 ^b^	−0.11 ± 0.02 ^b^	−1.60 ± 0.03 ^b^	3.78 ± 0.09 ^a^
UEL-MRPs	−3.53 ± 0.02 ^c^	−0.08 ± 0.01 ^ab^	−1.53 ± 0.02 ^a^	3.85 ± 0.02 ^a^

Note: Results were expressed as mean value ± standard deviation (*n* = 3). Values bearing different letters (a to c) were significantly different (*p* < 0.05).

**Table 3:** Volatile compounds in the MRPs.

No.	Volatile Compounds	^1^ KIs	^2^ Odors	Relative Concentration [ng kg^−1^] (Mean ± SD)			
				N-MRPs	UN-MRPs	EL-MRPs	UEL-MRPs
	Aldehydes (7)			16,325.90 ± 2633.65 ^b^	23,312.20 ± 2256.92 ^b^	42,201.86 ± 6487.71 ^a^	51,094.08 ± 6451.28 ^a^
1	2-undecenal	1311	waxy	----	----	----	1526.60 ± 439.78
2	nonanal	1104	fatty, citrus	2544.39 ± 789.48 ^c^	3150.21 ± 1627.73 ^c^	13,575.11 ± 744.44 ^a^	8164.12 ± 1311.46 ^b^
3	octanal	92	fatty, citrus, honey	----	628.01 ± 58.54 ^c^	5827.26 ± 424.38 ^a^	2740.49 ± 624.90 ^b^
4	benzaldehyde	982	almond	13,383.74 ± 2478.88 ^b^	18,797.82 ± 385.02 ^b^	21,529.94 ± 5270.65 ^a^	38,662.87 ± 8787.69 ^a^
5	4-methoxy-benzaldehyde	1171	hawthorn	----	214.49 ± 59.06	----	----
6	decanal	1204	fatty, sweet orange	397.76 ± 65.39 ^b^	521.67 ± 154.22 ^b^	868.86 ± 65.75 ^a^	----
7	(E)-2-octenal	1013	----	----	----	400.68 ± 178.09	----
	Ketones (4)			500.64 ± 33.39 ^b^	592.36 ± 40.27 ^b^	635.48 ± 117.50 ^b^	4302.12 ± 1105.77 ^a^
8	2H-pyran-2,6(3H)-dione	1098	----	91.11 ± 20.19 ^b^	135.62 ± 13.81 ^b^	140.31 ± 40.39 ^b^	386.13 ± 76.23 ^a^
9	acetoin	717	buttery	112.13 ± 23.42 ^c^	106.88 ± 20.41 ^c^	495.16 ± 77.50 ^b^	756.99 ± 160.41 ^a^
10	1-hydroxy-2-propanone	698	----	297.40 ± 9.22	----	----	3159.00 ± 875.38
11	6-methyl-5-hepten-2-one	938	fatty, green, citrus-like	----	349.86 ± 48.08	----	----
	Alcohols (11)			4696.10 ± 431.42 ^d^	10,404.52 ± 1020.19 ^c^	13,917.84 ± 1352.76 ^b^	37,637.68 ± 2448.57 ^a^
12	2-furanmethanol	885	burnt, caramel	951.36 ± 27.19 ^b^	1242.79 ± 330.92 ^b^	2623.38 ± 348.82 ^a^	1000.58 ± 152.83 ^b^
13	1-pentanol	761	----	----	621.58 ± 175.56 ^c^	4269.34 ± 411.52 ^b^	9280.11 ± 316.91 ^a^
14	1-octen-3-ol	969	mushroom	----	----	----	7635.86 ± 1535.68
15	2-methyl-3-pentanethiol	793	----	----	2140.45 ± 320.99	----	1730.77 ± 342.90
16	1-hexanol	860	green, fruity	1106.16 ± 39.61 ^c^	2882.99 ± 293.59 ^b^	1959.74 ± 285.49 ^bc^	6247.56 ± 933.66 ^a^
17	1,4-butanediol	904	----	112.70 ± 27.90	----	----	424.19 ± 15.77
18	benzyl alcohol	1036	fruity	----	----	1134.57 ± 175.23	----
19	phenylethyl alcohol	1136	roses	529.12 ± 17.16	649.71 ± 92.40	----	----
20	1-heptanol	960	weak alcoholic	----	----	----	4115.44 ± 414.03
21	maltol	1063	caramel	1559.63 ± 345.82 ^c^	2331.50 ± 259.26 ^c^	3930.81 ± 601.33 ^b^	7203.18 ± 538.75 ^a^
22	1-dodecanol	1457	fatty	437.12 ± 145.13	535.49 ± 89.77	----	----
	Esters (3)			1413.30 ± 226.14 ^c^	1755.50 ± 99.63 ^bc^	2245.81 ± 515.19 m^b^	5204.68 ± 231.95 ^a^
23	butyrolactone	825	----	1003.15 ± 223.50 ^c^	1192.48 ± 212.87 ^c^	2245.81 ± 630.98 ^b^	3749.90 ± 193.20 ^a^
24	5-ethyldihydro-2(3H)-furanone	986	caramel	----	----	----	573.71 ± 44.52
25	hexadecanoic acid, methyl ester	1878	----	410.15 ± 12.07 ^b^	563.03 ± 154.90 ^b^	----	881.07 ± 9.14 ^a^
	Acids (9)			8519.72 ± 535.84 ^b^	10,217.13 ± 741.44 ^b^	15,040.33 ± 997.20 ^a^	14,338.67 ± 1294.21 ^a^
26	isovaleric acid	811	rancid	5806.91 ± 786.41 ^b^	5495.16 ± 592.05 ^b^	8090.93 ± 853.77 ^a^	7441.90 ± 54.91 ^a^
27	n-decanoic acid	1372	fatty, rancid	----	378.11 ± 26.06	721.44 ± 142.74	----
28	hexanoic acid	974	fatty, waxy,	1480.04 ± 399.09 ^c^	1781.70 ± 230.72 ^bc^	2802.08 ± 545.34 ^a^	2394.52 ± 288.82 ^ab^
29	octanoic acid	1173	waxy, fatty	476.11 ± 97.97 ^c^	1156.04 ± 155.08 ^b^	2114.29 ± 498.28 ^a^	2560.38 ± 451.35 ^a^
30	nonanoic acid	1272	----	264.56 ± 8.58 ^c^	378.17 ± 62.92 ^b^	461.28 ± 40.42 ^a^	485.55 ± 17.18 ^a^
31	heptanoic acid	1073	waxy, fruity, fatty	226.64 ± 24.61 ^c^	421.16 ± 68.00 ^b^	538.76 ± 96.81 ^b^	673.93 ± 65.34 ^a^
32	pentanoic acid	875	----	139.37 ± 23.12	606.79 ± 71.25	----	----
33	butanoic acid	811	rancid	----	----	311.54 ± 20.75	----
34	pentadecanoic acid	1869	waxy	126.09 ± 34.85	----	----	----
	Pyrazines (2)			----	756.89 ± 144.74	----	2235.33 ± 311.44
35	2,5-dimethyl-pyrazine	894	Cocoa, roasted, nutty	----	----	----	2235.33 ± 311.44
36	tetramethyl-pyrazine		nutty, chocolate, coffee	----	756.89 ± 144.74	----	----
	Furans (2)			600.96 ± 73.22 ^b^	842.49 ± 191.62 ^c^	2369.50 ± 398.75 ^a^	2565.21 ± 176.92 ^a^
37	bis(2-methyl-3-furyl) disulfide	1745	roasty, meat, sulfur	600.96 ± 73.22 ^b^	842.49 ± 191.62 ^a^	666.67 ± 91.68 ^ab^	524.40 ± 94.07 ^b^
38	1-(2-furanyl)-Ethanone	878	almond, nut, roasted	----	----	1702.83 ± 329.22	2040.81 ± 89.87
	Hydrocarbons (3)			----	678.87 ± 208.19 ^ab^	1047.89 ± 327.45 ^a^	314.59 ± 50.54 ^b^
39	n-hexane	618	gasoline	----	----	----	314.59 ± 50.54
40	nonadecane	1910	----	----	678.87 ± 208.19	----	----
41	pentadecane	1512	----	----	----	1047.89 ± 327.45	----
	Phenols (4)			1094.01 ± 59.50 ^d^	2181.28 ± 357.30 ^c^	6203.81 ± 445.34 ^b^	7909.26 ± 221.45 ^a^
42	butylated hydroxytoluene	1668	----	----	----	3796.72 ± 348.50	3963.23 ± 502.73
43	phenol	901	----	395.39 ± 30.41 ^c^	378.72 ± 92.58 ^c^	636.09 ± 131.79 ^b^	1090.62 ± 146.61 ^a^
44	p-cresol	1014	----	----	526.95 ± 189.71	----	695.62 ± 166.81
45	2-methoxy-phenol	1090	----	698.62 ± 65.46 ^d^	1275.61 ± 247.70 ^c^	1770.99 ± 41.55 ^b^	2159.78 ± 92.60 ^a^
	Thiazoles (1)						
46	4-methyl-5-thiazoleethanol	1264	meaty, roasted	1582.85 ± 236.87 ^c^	2018.86 ± 74.48 ^c^	4492.16 ± 215.17 ^b^	8787.13 ± 1646.45 ^a^
	Ethers (1)						
47	3-tert-butyl-4-hydroxyanisole	1417	----	----	----	752.29 ± 76.05	742.80 ± 18.10
	Pyrroles (1)						
48	1-(1H-pyrrol-2-yl)-ethanone	1035	walnuts, toast	237.32 ± 5.26 ^d^	477.30 ± 83.31 ^c^	665.75 ± 10.75 ^b^	1288.37 ± 146.92 ^a^
	Thiophenes (1)						
49	5-methyl-2-thiophenecarboxaldehyde	1072	almond, fruity, nutty	----	----	----	873.56 ± 95.92

Note: Means bearing different letters are significantly (*p* < 0.05) different in the same line. “----”, not detected. ^1^ KI (Kovats indices) determined by searching the mass spectrum in the database and manual interpretation. ^2^ Odors indicated the odor bias of some specific flavor compound.

**Table 4:** Sensory evaluation scoring criteria of MRPs.

Sensory Indicators	Judging Controls	Scoring Criteria/Point
Off-flavor	The unaccepted flavor of rotten eggs, prepared by putting broken eggs (100 g) at 50 °C for 7 days, was used as odor intensity evaluation.	Strong odor: 0–2
		Medium odor: 2–5
		Lighter odor: 5–7
		Odorless: 7–10
Meaty	Take certain pork lean meat, cut into 2.5 cm cubes, cook in water for 2 h, and then use as a meat flavor evaluation control.	Strong odor: 7–10
Umami	The umami used sodium glutamate solution (1%, *w*/*v*) as the umami note.	Medium odor: 5–7
Salty	Salty taste is the taste of 0.5% (*w*/*v*) sodium chloride solution.	Lighter odor: 2–5
Total acceptance	Evaluation based on meaty, umami, salty, and off-flavor.	Odorless: 0–2

Note: The score was given on a scale of 0 (undetected) to 10 (strong). The sensory evaluation standard of off-flavor is opposite to other indexes.

## Data Availability

The data that support the findings of this study are available from the corresponding author upon reasonable request.
